# Design and test of a MEMS strain-sensing device for monitoring artificial knee implants

**DOI:** 10.1007/s10544-013-9770-z

**Published:** 2013-05-11

**Authors:** W. Hasenkamp, N. Thevenaz, J. Villard, A. Bertsch, A. Arami, K. Aminian, A. Terrier, P. Renaud

**Affiliations:** 1 École Polytechnique Fédérale de Lausanne—EPFL, 1015 Lausanne, Switzerland; 2 Microsystems Laboratory—LMIS4, Lausanne, Switzerland; 3 Laboratory of Movement Analysis and Measurements—LMAM, Lausanne, Switzerland; 4 Laboratory of Biomechanical Orthopedics—LBO, Lausanne, Switzerland; 5 Neurocentre—Centre du dos, Avenue de Savoie 10, 1003 Lausanne, Switzerland

**Keywords:** Load monitoring, Biosensor, Strain sensor, Total joint arthroplasty

## Abstract

This paper describes the development of a polyimide-based MEMS strain-sensing device. Finite element analysis was used to investigate an artificial knee implant and assist on device design and to optimize sensing characteristics. The sensing element of the device was fabricated using polyimide micromachining with embedded thin-metallic wires and placed into a knee prosthesis. The device was evaluated experimentally in a mechanical knee simulator using static and dynamic axial load conditions similar to those encountered *in vivo*. Results indicates the sensor is capable of measuring the strain associated to the total axial forces in the range of approximately 4 times body weight with a good sensitivity and accuracy for events happening within 1 s time window.

## Introduction

Total Knee Arthroplasty (TKA) is a widely used surgical procedure to replace a damaged knee joint by an artificial knee implant (Kirking et al. 2006). Due to the increase in population and life time expectancy the number of TKA surgeries has been continuously increasing. Moreover the age at the time of primary TKA is decreasing therefore it is critical to ensure operation success and to access the status of the artificial knee implant along its lifetime to minimize the possibilities of revision surgery and to maximize the longevity of the implant (Heinlein et al. 2009).

The knee is a complex joint that is difficult to model accurately, however mathematical models are commonly used as initial screening tools for evaluating the prosthesis design. Finite Element Analysis (FEA) is the most frequently used technique to evaluate the artificial implants, mainly to investigate the influence of load application and identify fragile regions to avoid premature prosthesis failure (Bergmann et al. 2008). If FEA contributes to extend the life of the orthopedic implant other factors significantly impact on the prosthesis lifetime. Several different artificial knee implant designs are commercially available but misalignment, leading to knee imbalance, and wearing are still the major reasons for revision. Forces acting directly on the artificial joint affect the knee balance and induce wear of the bearing surface, which is associated to prosthesis loosening, consequently impacting on the implant lifetime (D’Lima et al. 2006).

One of the most affected components of the artificial knee implant is the ultra-high-molecular-weight polyethylene (UHMWPE) insert, due to its geometry and the high forces acting upon it (Arami et al. 2011). Therefore monitoring the strain, associated to knee imbalance and forces acting upon the prosthesis, can help on the development of new articulating components, lead to a better understanding of the artificial knee biomechanics, support improvements on the mathematical models that describes the constitutive model of the materials and the knee behavior, improve prosthesis alignment during surgery and give continuous feedback on the status of the artificial knee implant.

Valuable efforts have been made to design implantable systems for monitoring biomedical implants, either using strain gauges, fiber Bragg gratings or Tekscan sensing systems (Taylor et al. 1998; Kirking et al. 2006; Heinlein et al. 2007; Mohanty et al. 2007). Though the systems have their specific advantages many require alterations of the current prosthesis designs or can only be used during surgery not being suitable for implantation. To overcome this limitations sensors can be fabricated using biocompatible materials, such as polyimide, and embedded into the polyethylene insert without introducing design changes (Crescini et al. 2009, 2011). Polymer-based microelectromechanical systems (MEMS) are increasingly being used in biomedical applications (Grayson et al. 2004) and, recently, micro-machined polyimide sensors have been used as sensing elements in a broad range of biomedical applications, e.g. deep brain recording and stimulation (Mercanzini et al. 2008) and contact lens pressure sensors for glaucoma (Leonardi et al. 2009).

In this paper we present a versatile MEMS strain-sensing device for the monitoring of loads acting upon an artificial knee implants, at the level of the UHMWPE insert. The goal of evaluating the strain is to help surgeons on the alignment of prosthesis, which can improve the knee balance and provide a follow up tool to help monitoring the artificial knee along its lifetime assuring the overall surgery quality. Likewise, the strain monitoring, which is associated to loads acting upon the prosthesis, can lead to a better understanding of the artificial knee biomechanics and help on the development of new generation of implants. Moreover, the continuous monitoring of the strain evolution can be used to track the wear of the UHMWPE insert. The basic design and working principle of the sensors are presented as well as results of the preliminary bench tests. The manufacturing process is based on polyimide micro-machining, which allows to adapt the shape and design of the micro-devices. The sensors are based on polyimide-metal-polyimide sandwich structures that are embedded into the UHMWPE part.

## Finite element analysis

**Fig. 1:**
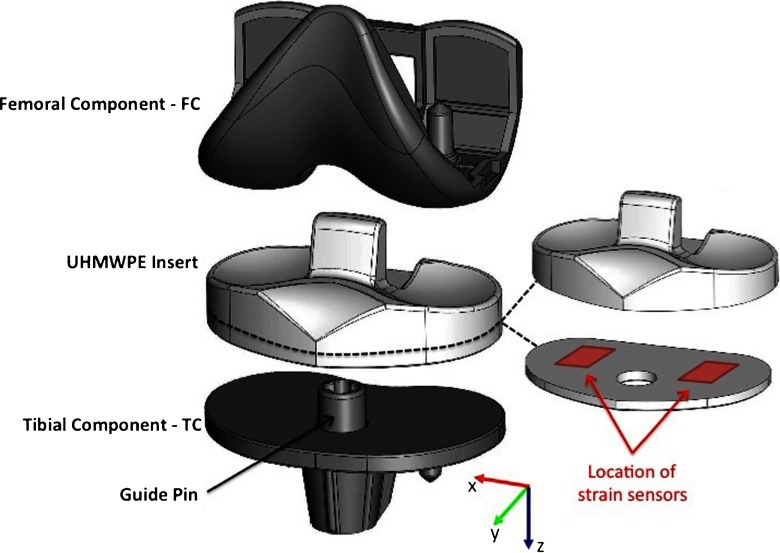
Components of the CAD model comprising the femoral component (FC), the UHMWPE insert, the tibial component (TC) and the guide pin, and a UHMWPE insert cross-section depicting the location of the strain sensors

**Fig. 2:**
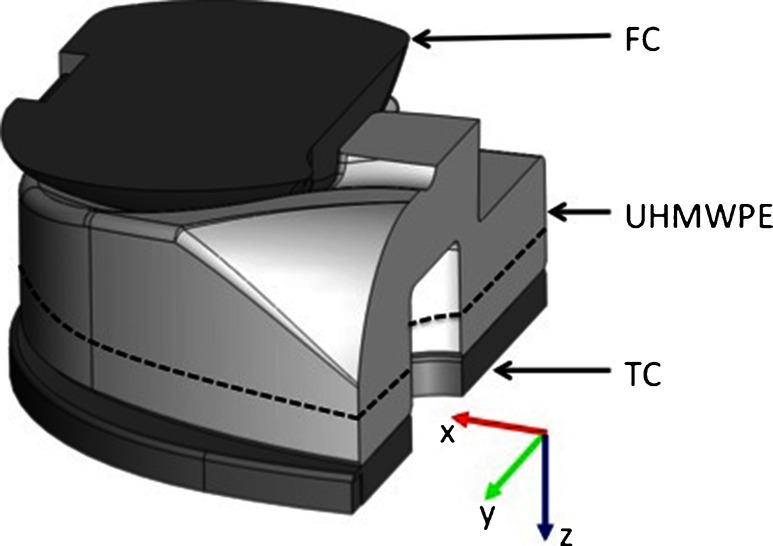
Simplified 3D CAD model used in the FEA

Finite Element Analysis (FEA) is an effective tool to investigate the distribution of the stress and strain in various kinds of engineering structures (Kasi et al. 2011). In this article, FEA was used to investigate the distribution of strain in an artificial knee implant and assist on the strain gauges placement inside the ultra-high-molecular-weight polyethylene insert (UHMWPE). The Computer Assisted Design (CAD) model was obtained from the manufacturer of an artificial knee, SYMBIOS Orthopédie SA (Yverdon-Les-Bains, Switzerland), and the 3D finite element model built into a commercial FEA software, COMSOL Multiphysics (v4.2). The components of the CAD model is presented in Fig. 1, and comprises the femoral component (FC), the UHMWPE insert, the tibial component (TC) and the guide pin. The location of the strain sensors in a cross-sectioned UHMWPE insert is also depicted in Fig. 1. The original CAD description of the prosthesis was simplified, without loss of (relevant) detail, in order to generate a 3D model suitable for computing. The simplified 3D model is sketched in Fig. 2.

FEA was modeled in the structural mechanics module of COMSOL in stationary mode assuming a linear elastic behavior for all parts. The fundamental considerations of this approach are to assume a small strain (or stress) and the linear relationship between components stress/strain (i.e. Young’s modulus). The material properties (Young’s modulus *E* and Poisson constant $\nu $) used in the FEA are the following: for the FC and TC (made of a Co-Cr-Mo alloy) $E=115$ GPa, $\nu =0.3$, and for the UHMWPE $E=0.34$ GPa, $\nu =0.4$. These values were suggested by the prosthesis manufacturer.

To establish boundary conditions for the simulation the following constraints were added to the model. Between the FC and the UHMWPE insert a contact interface is established. A surface constraint is defined on the guide pin hole to keep the alignment between the UHMWPE insert and the TC. Boundary condition uniaxial loads acting on the prosthesis were applied to the upper flat area of the FC, in the z-axis direction, varying from 200 N up to 3100 N, and defined as such to be in accordance with the biomechanical conditions in the human body. To complete the model, a fixed constraint is defined at the bottom surface of part of the TC to avoid overall implant displacement which can introduce discrepancies in the simulations. An unstructured progressive triangular meshing algorithm, to form tetrahedral elements, was utilized for meshing the model. The minimum and maximum element size was defined to 0.1 mm and 1 mm, respectively. The meshed structure consisted of 116933 tetrahedron elements and the convergence criteria for simulations was established by a MUMPS solver. FEA was used to investigate the distribution of strain inside the UHMWPE insert and identify regions for placement of strain gauges sensors. In order to comply with implants regulatory standards the sensors where placed in the xy-plane at 6 mm from the FC/UHMWPE bearing surface (refer to Fig. 1).

## Results of finite element analysis

**Fig. 3:**
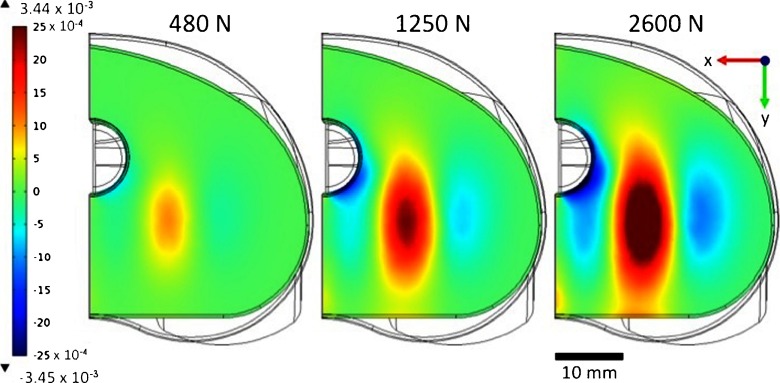
Evolution of the x-component strain, in the xy-plane at 6 mm from the FC/UHMWPE bearing surface, for different applied loads

Figure 3 shows the evolution of the x-component strain, in the previously defined xy-plane (at 6 mm from the FC/UHMWPE bearing surface), for different applied loads. Highly positive strain values, associated with tensile strain in the x-axis direction, are visible under the contact points of the FC/UHMWPE bearing surface. Negative strain values, associated with compressive strain in the x-axis direction are also visible on the surroundings of the tensile region. Moreover, from this investigation we could identify a xz-plane with good strain symmetry at nearly 1/3 of the UHMWPE height, therefore these region is suitable for positioning the sensors.

**Fig. 4:**
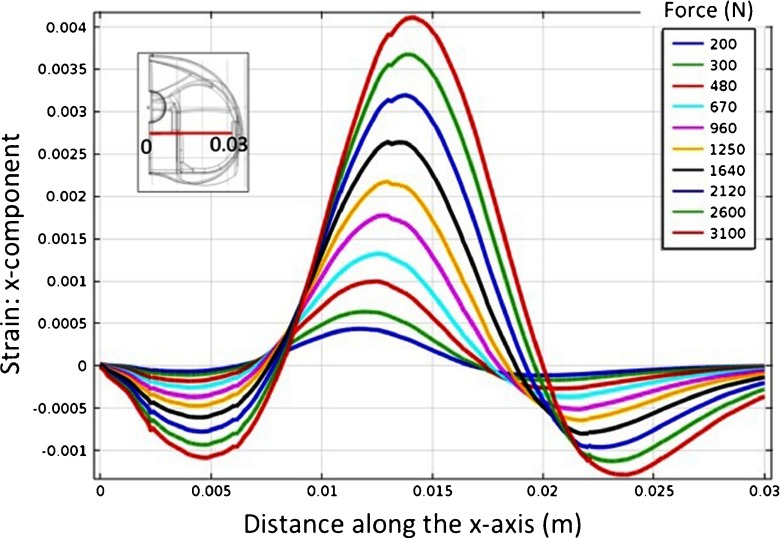
Evolution of the x-component strain at the line of intersection between the xy- and the xz-planes, along the UHMWPE width, for different applied loads

At the intersection between the defined xy- and xz-plane a line is defined and used to position and orient the strain sensors. Figure 4 presents the evolution of the x-component strain at the intersection of xy- and xz-planes, along the UHMWPE width, for different applied loads. The insert in Fig. 4 shows the line of intersection between the xy- and xz-planes where the strain values were taken. Highly compressive strain is visible in a region that extends roughly for 6 mm, and having its maximum nearly 13 mm along the UHMWPE width. Therefore, the strain sensors were defined to be located in the compressive strain region with is center located at 13 mm along the UHMWPE insert width, while passive strain sensors will be located in regions of zero strain to compensate for overall temperature variations.

## Sensor design, fabrication and packaging

**Fig. 5:**
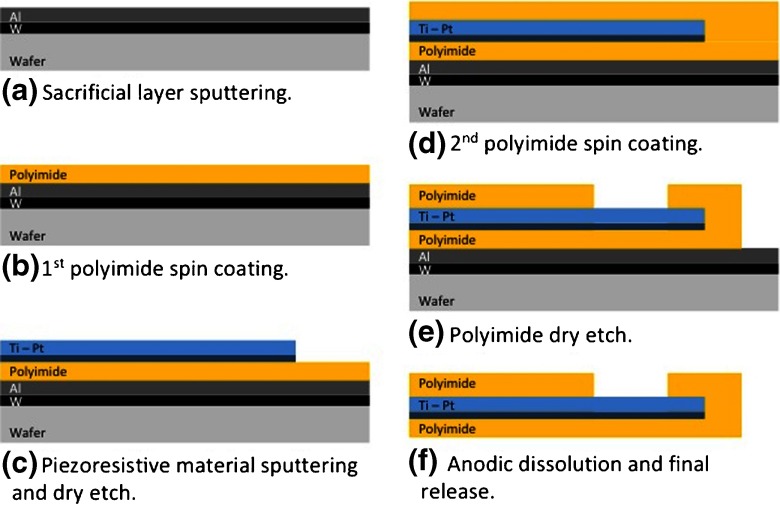
Cross section view of the fabrication process

The strain sensors resistance was defined to be 3.2 kΩ in order to decrease power consumption and facilitate readout. The strain sensors to be embedded into the UHMWPE were built in polyimide-metal-polyimide sandwich structures by dry etching, using standard photolithography manufacturing processes (Mercanzini et al. 2008; Leonardi et al. 2009). Polyimide (PI) is an excellent material for biomedical microdevices due to its chemical and thermal stability, low water uptake and biocompatibility (Richardson et al. 1993). Such PI properties are crucial because the sensors are placed under bearing surfaces which are prone to wear and it will not risk patients health. Furthermore, PI is widely used in integrated circuit manufacturing, therefore suitable for mass production. The total thickness of the sensor is about 10 μm. A cross-sectional view of the microfabrication process is presented in Fig. 5.

The detailed microfabrication process comprises the following steps: A sacrificial layer of tungsten (100 nm) and aluminum (1 μm) is first evaporated onto a carrier silicon wafer (Fig. 5a). A 5 μm layer of PI (PI2611, HD Microsystems) is applied on top of the aluminum by spin-coating and cured at 300 °C for 1 h in nitrogen atmosphere (Fig. 5b). A titanium adhesion layer (20 nm) and platinum layer (180 nm) are then sputtered onto the cured polyimide. The strain gauges are patterned by reactive ion etching in Cl$_{2}$ using a patterned photoresist as an etch mask (Fig. 5c). A second layer of PI, 5 μm in thickness, is spin-coated and likewise cured (Fig. 5d). An etch mask of sputtered SiO^2^ (500 nm) is deposited onto the sandwich structure and then patterned by reactive ion etching using a photoresist etch mask. This oxide layer is then used as hard mask during the subsequent oxygen-plasma etch of the polyimide to define both the structure outline and open contact pads to the strain gauges (Fig. 5e). The polyimide devices are detached from the silicon carrier wafer by anodic metal dissolution in a 10 wt % sodium chloride solution: the substrates are immersed in the salt solution at room temperature with a platinum counter electrode, and a constant positive potential (0.7 V) is applied to the aluminum layer. The anodic metal dissolution process dissolved the aluminum, leaving the tungsten on the substrate and releasing the polyimide-metal-polyimide structures (Fig. 5f).

**Fig. 6:**
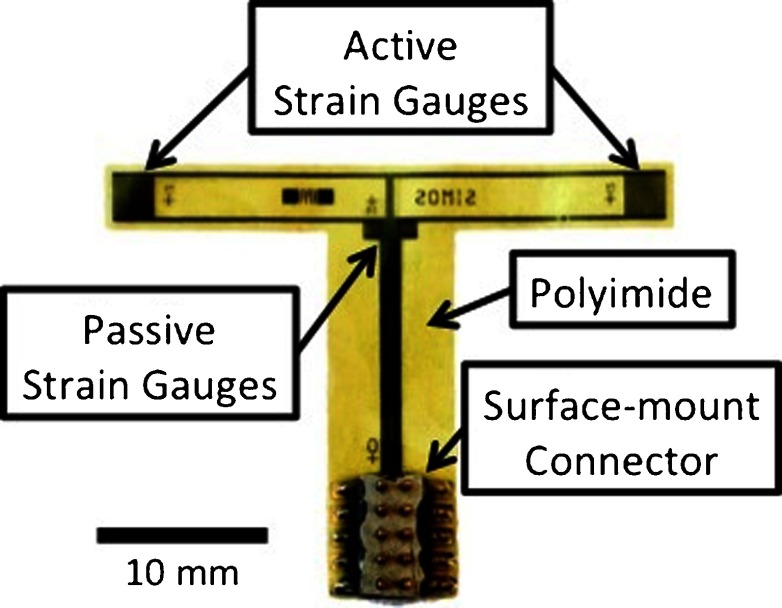
Polyimide-metal-polyimide micro-machined structure

The packaging of the device consists of bonding a surface-mount connector (Samtec, Inc.) to the contact pads using a conductive epoxy. The connector provides the link between the strain gauges and the electronic circuitry. Despite the utilization of a connector prevents the usage of this device *in vivo*, the technology versatility allows for design changes without prejudicing the device feasibility. A fabricated polyimide-metal-polyimide structure is shown in Fig. 6. It consists of four strain gauges, two active gauges positioned (according simulation results) under the contact bearing surfaces, and two passive gauges towards the center of the artificial knee in regions of zero stain (according simulations).

**Fig. 7:**
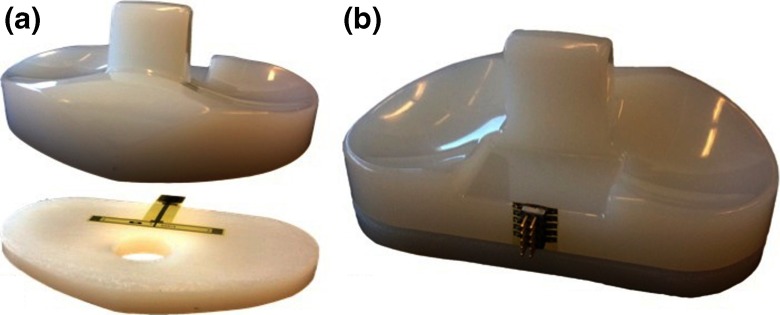
**a** A cross-sectioned UHMWPE insert with the strain sensors positioned for final assembly and (**b**) the complete packaged device re-joined and sealed using a biocompatible epoxy glue

To complete the fully packaged strain-sensing device the sensors are embedded into the UHMWPE insert. For that, the UHMWPE insert is sectioned in two parts, the polyimide-metal-polyimide sandwich is positioned, and the UHMWPE parts re-joined and sealed using a biocompatible epoxy glue. A cross-sectioned UHMWPE insert with the strain sensors positioned for final assembly is presented in Fig. 7a and the complete packaged device is shown in Fig. 7b. The packaged device is capable of continuous and real-time measurement. The built strain-sensing devices are versatile, simple, cost effective, and are ready to be integrated with implantable wireless telemetry, which can increase monitoring efficiency outside healthcare facilities.

## Experimental setup and results

The experimental study is carried out in a mechanical knee simulator (MTS bionix servohydraulic test systems). The tests are performed using static and dynamic axial load conditions (perpendicular to the referred xy-plane) similar to those encountered *in vivo*. The strain-sensing device is attached to the knee simulator and subjected to loads varying from 200 N up to 3100 N. The applied forces are close-loop controlled by a load cell, attached underneath the strain-sensing device, and connected to the knee simulator controller.

The fully packaged strain-sensing device is connected in a Wheatstone bridge configuration using external standard resistors with similar impedance to those in the polyimide-metal-polyimide sandwich. The bridge is DC powered with 2.5 V. The output signals are recorded with a National Instrument acquisition board (NI-Daqpad-6015) through a signal conditioning unit (SC-2345) connected to a full-bridge input channel (SCC-SG04). For displaying and recording the measurements a LabView (National Instruments) interface is configured. The signal conditioner’s gain and span controls for the strain-sensing devices are set to obtain a full-scale electrical output signals.

**Fig. 8:**
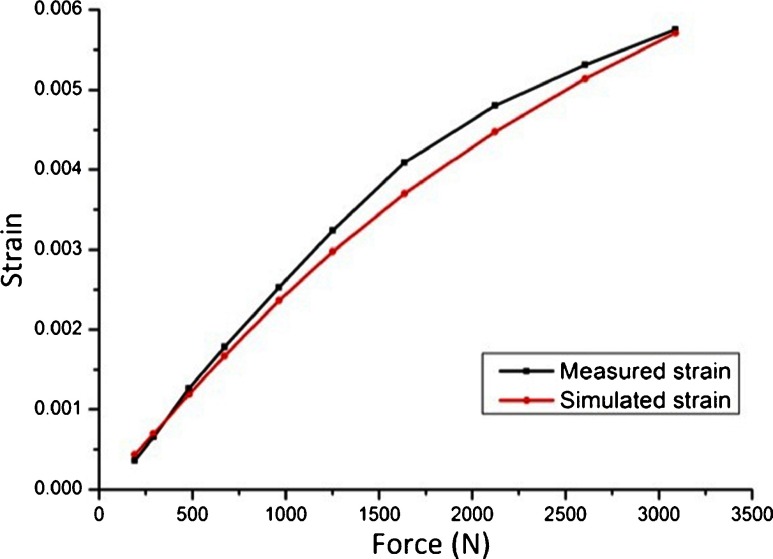
Simulated x-component strain and measured strain as a functions of applied loads

Results for the identification (fitting) of parameters of the strain-sensing device are presented in Fig. 8. The curve presents both, the expected strain (simulated x-component strain) and the measured strain as a functions of applied loads. The measured strain was calculated from the output voltage of the Wheatstone bridge. In total one insert was tested, meaning two separate active strain sensors, and each separate strain sensor was subject to the same applied load for at least 10 times. The error bars were included in Fig. 8 but they are of similar size as of the black squares that represents the measurements. These results indicates the measured strain is in accordance with the simulated strain, and that applied forces can be estimated from measured strain. Therefore, validating the results obtained from the numerical model. Despite the overall non-linear behavior of the device, the measured data presents a linear region within forces varying from 0 N up to 1500 N. This are level of forces exerted upon the knee during normal walking activities. A linear regression in this range of forces (from 0 N to 1500 N) indicates an average device sensitivity of 2.4 μ V/N (adjusted R-square = 0.99).

**Fig. 9:**
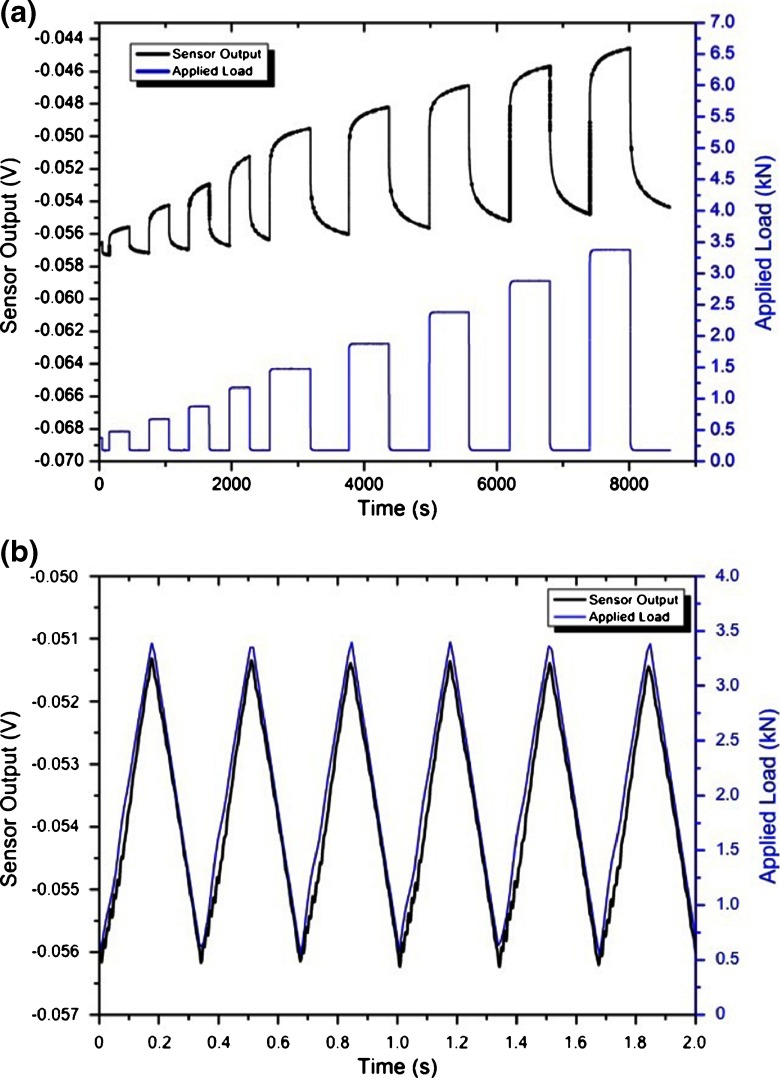
Series of slow (**a**) and fast (**b**) dynamic loading/unloading and respective sensor’s output as a function of time

Tests of the assembled device were also carried out dynamically. Figure 9 shows a series of slow (Fig. 9a) and fast (Fig. 9b) dynamic loading/unloading and the sensor’s output as a function of time. The influence of the UHMWPE viscoelastic behavior on the measurements can be verified on Fig. 9a, where non-linearities, i.e. creep, can be observed in the measurements. Creep has an undeniable influence on the repeatability of the measurements affecting device accuracy in long-term measurements. A good sensor response to fast dynamic loading can be verified in Fig. 9b, thus allowing the sensor to be used for measuring knee forces during walking.

**Fig. 10:**
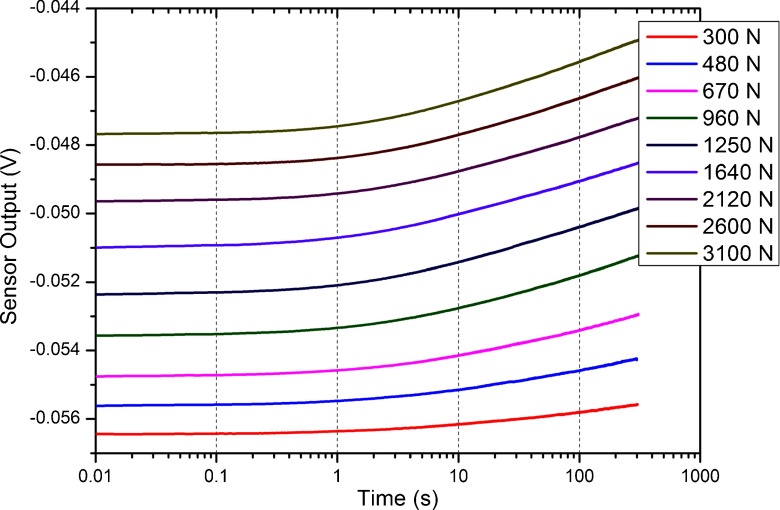
Experimental creep curves for different loading levels versus the logarithm of time

To analyze the influence of the creep on the measurements the experimental creep curve was extracted from the data. Figure 10 shows the experimental creep curves for different loading levels. The creep is plotted versus the logarithm of time. The experimental curves indicates the creep is dependent on the applied load as well as on the time. When analyzing Fig. 10 we are able to determine the proposed sensor is accurate for measuring events happening within 1 s time window, with errors below 8 % the applied load. For measurements lasting 10 s and 100 s the creep introduces substantial errors on the measurement, respectively, 38 % and 85 % the applied load. However, in slow pace walking a single load cycle is within a 1 s time window, therefore in gait studies it is important to monitor events happening bellow a 0.1 s time window, region where errors are bellow 3 %.

The developed device has thus the potential measure *in vivo* activities and help to improve the TKA surgery and postoperative followup. In TKA surgery the device can be used to assist on the ligament balance, which is currently only qualitatively assessed, and is crucial for the stability and lifetime of implants. During the postoperative physical therapy the device can provide information regarding the artificial knee implant function and help to improve overall rehabilitation and treatment of patients with total knee implants.

## Discussion

In the present study, a polymer-based strain-sensing device was developed for monitoring an artificial knee implant. The experimental data demonstrates the sensor is capable of measuring the strain associated to the total axial forces exerted on the UHMWPE insert in the range of approximately 4 times body weight with a good sensitivity and accuracy for events happening within 1 s time window. This device has been designed to monitor the total load and simple alterations on the strain-sensor design can allow the measurements of forces in each individual compartment of the UHMWPE insert, allowing for the monitoring of instantaneous artificial knee balance. Other alterations can be also made on the design to allow the measurement of other force components (e.g. momentum) and temperature monitoring, to investigate the effect of different activities, such as walking and cycling, on the implant wear rate. Improvements on the sensors design and packaging, as well as increasing the number of strain-sensors on the device can allow the mapping of strain in other regions of interest in the UHMWPE.

Despite, *in vivo* measurements of the knee joint forces are broadly available, very little is know on the evolution of artificial knee implants after TKA and the aging of such prosthesis, hence the proposed device will allow studies of biomechanics on the prosthetic knee and improve implants design. The results demonstrates the proposed strain-sensor represents a promising new system for *in vivo* load monitoring of medical implants with the advantage that this device can be introduced into the surgery procedure with minimal disruption to usual protocols. Also we expect this sensor could be used to investigate the creep deformation on the UHMWPE. Creep is a property of viscoelastic materials (Phan 2008) which introduces non-linearities on the system and are a cause for errors in long-term measurements. The understanding of the creep behavior of the UHMWPE can also help on accurate quantify the prosthesis wear (El-Domiaty and El-Fadaly 2002).

The strain-sensing device is simple to package and represents a cost effective solution since it does not imply changes on current artificial knee implant designs. Future applications include the ability to validate mathematical models that describes the knee biomechanics, the capability to study the effect of postoperative physical therapy and evaluate the knee balance to improve overall rehabilitation. Moreover, it can be customized for different models of prosthesis combined with various materials, therefore the proposed autonomous sensor can lead to new redesigns regarding the function of knee implants and the treatment of patients with total knee implants. Additionally, the device could be integrated with low-power wireless telemetry, which will allow for long-term measurements *in vivo*.

## Conclusion

In this work, we demonstrated a polyimide-based MEMS strain-sensing device for monitoring knee implants. Throughout the design process, FEA modeling results were used to optimize device placement inside an artificial knee component (UHMWPE). The PI-based technology is well suited for biomedical applications and can provide a significant cost advantage since it does not implies changes on current prosthesis. The device was subjected to tests in a mechanical knee simulator using static and dynamic axial load conditions similar to those encountered *in vivo*. Results indicated the measured strain is in accordance with simulated strain and that the applied forces can be estimated from measured strain. The experimental data demonstrates the sensor is capable of measuring the strain associated to the total axial forces in the range of approximately 4 times body weight with a good sensitivity and accuracy for events happening within 1 s time window.
